# Detection of intravenous infiltration using impedance parameters in patients in a long-term care hospital

**DOI:** 10.1371/journal.pone.0213585

**Published:** 2019-03-21

**Authors:** Ihn Sook Jeong, Eun-Joo Lee, Jae Hyung Kim, Gun Ho Kim, Young Jun Hwang, Gye Rok Jeon

**Affiliations:** 1 College of Nursing, Pusan National University, Yangsan, Republic of Korea; 2 Dept, of Nursing, Dong-Eui University, Busan, South Korea; 3 Research Institute of Nursing Science, Pusan National University, Yangsan, Republic of Korea; 4 Dept. of Medical Science, School of Medicine, Pusan National University, Yangsan, Republic of Korea; 5 Dept. of Biomedical Engineering, School of Medicine, Pusan National University, Yangsan, Republic of Korea; University Magna Graecia of Catanzaro, ITALY

## Abstract

This study was aimed to evaluate the changes of impedance parameters of patients who were admitted to a long-term care hospital by measuring bioelectrical impedance. The subjects were 18 patients who had infusion therapy through peripheral intravenous (IV) catheters and had at least an infiltration. The impedance parameters were measured with a multi-channel impedance measuring instrument (Vector Impedance Meter) twice; at starting IV infusion after catheter insertion and infiltration detected. As results, the resistance (*R*) after infiltration significantly decreased compared to the initial resistance. At 50 kHz, the resistances were 498.2±79.3 [Ω] before infiltration and 369.4±85.6 [Ω] after infiltration. The magnitude of the reactance (*X*_*C*_) decreased after infiltration. At 50 kHz, the measured reactance was -31.1±8.3 [Ω] before infiltration and -24.5±5.9 [Ω] after infiltration. The data points plotted in the *R-X*_*C*_ graph shifted from the first quadrant before infiltration to third quadrant after infiltration. Our findings suggest that bioelectrical impedance is an effective method for detection of infiltration in a noninvasive and quantitative manner.

## Introduction

Infiltration is one of the most common health complications in infusion therapy involving a peripheral intravenous (PIV) catheter [1]. Patients with infiltration often experience pain and skin damage, and can even a persistent disability of the arm [2]. Thus, infiltration can significantly impact the patient’s wellbeing and must be carefully monitored at an early stage [3]. Infiltration is known to be difficult to detect particularly during its initial stages. To date, infiltration has relied primarily on clinical and visual methods to examine skin and tissues surrounding the IV catheter site for factors such as tissue pressure, color, edema, swelling and temperature. However, these methods are disadvantageous because infiltration can only be confirmed well after infiltration has progressed considerably or tissue damage has occurred in subcutaneous tissue. As recognition of early signs and symptoms of infiltration can effectively limit the amount of fluid that leaks from the vein into the subcutaneous tissue [4], several infiltration detection systems have been introduced.

Optical methods near infrared (NIR) light have been developed to facilitate noninvasive monitoring of IV sites [5,6]. High-resolution variable frequency ultrasound imaging is increasingly being used in the noninvasive evaluation of various cutaneous diseases [7]. Ultrasound could detect small volumes of fluids such as cosmetic fillers and subcutaneous injections subcutaneous injections and thus provides a potential reference standard for the future evaluation of IV monitoring device [8]

The infiltration detection system using bioelectrical impedance analysis (BIA) can be another option [9]. BIA has been employed to diagnose diseases [10] and to assess the hydration status, body composition, muscle–fat ratio, obesity degree, lean balance, edema, and nutritional status of the patients [11,12]. As increase in local conductive IV fluids is known to reduce the measured impedance [13], fluid leakage due to infiltration can increase the skin firmness which leads to change in bioelectrical impedance around the IV catheter site [9]. Therefore, we have investigated changes of impedance parameters such as impedance, resistance, reactance, and capacitance between pre and post infiltration, as the IV solution accumulates in the subcutaneous tissues and cell membrane functions in rabbits [14] and pigs [15]. When IV solution was properly infused into the vein on the back of rabbit, the impedance parameters (impedance, resistance, reactance, and capacitance) measured at five injection sites showed almost similar behavior with very slight standard deviations. On the other hand, the impedance parameters were significantly different before and after infiltration [14]. In addition, when infiltration was intentionally induced in the vein of a pig's posterior ear, impedance parameters (resistance, reactance, and capacitance) showed significant differences before and after infiltration [15].

Based on the preclinical studies, this study was aimed to evaluate the impedance parameters of patients at a long-term care hospital by measuring bioelectrical impedance. This is a preliminary study aimed to develop a point-of-care device and an automated impedance device that can detect infiltration in the nursing field.

## Materials and methods

### Study subjects

The subjects of this prospective observational study were 18. who met the following inclusion criteria; patients who had infusion therapy through PIV catheters from July 18th to September 7th, 2017, had at least an infiltration, and gave written informed consent by themselves or their legally authorized representatives (LARs). There were 3 males and 15 females, and their mean age was 79.5 years, ranged 57–87 years. Mean diameter of infiltration was 2.3cm, ranged 0.4-7cm. Table 1 shows the more characteristics of subjects.

**Table 1:** The characteristics of subjects.

id	Gender	Age	Measure site	Infiltration diameter(cm)	Type of Infusate	
					BI^a^	AI^b^
1	F	87	heel	3	N/S^c^	N/S^c^
2	F	83	heel	1	10% DW^d^	10% DW^d^
3	F	81	forearm	3	N/S^c^	N/S^c^
4	F	76	dorsum of hand	1	H/S^e^	H/S^e^
5	F	87	upper arm	3	H/D^f^	H/D^f^
6	M	57	dorsum of foot	7	Combiflex	N/S^c^
7	F	74	forearm	1	H/S^e^	H/S^e^
8	F	86	heel	2	5% DW^d^	5% DW^d^
9	F	85	forearm	3	H/D^f^	H/D^f^
10	F	87	forearm	3	H/D^f^	H/D^f^
11	M	74	ankle	2	N/S^c^	N/S^c^
12	M	82	lower leg	0.5	H/D^f^	H/D^f^
14	F	81	forearm	2	5% DW^d^	5% DW^d^
15	F	86	dorsum of foot	0.7	H/D^f^	H/D^f^
16	F	79	dorsum of foot	0.4	H/D^f^	H/D^f^
17	F	81	thigh	3	5% DW^d^	5% DW^d^
18	F	64	lower leg	2	N/S^c^	N/S^c^

^a^BI: before infiltration

^b^BI: before infiltration

^c^N/S: normal saline

^d^DW: dextrose water

^e^H/S: Hartmann solution

^f^H/D: Hartmann dextrose

### Study instrument

Multi-channel impedance measuring instrument (Vector Impedance Meter) developed by Kim et al. for detection of infiltration is as follows [16]. A weak alternating current (800 μA) and multiple frequencies (10 kHz~10 MHz) are applied to the human body to measure impedance parameters (resistance: *R*, reactance: *X*_*C*_, phase angle: *θ*) in the live body. Radio frequency (RF) weak current is applied to the electrode located in the periphery of the injection site and the impedance and phase change of the arbitrary points among the multiple channels or specified channels.

### Study procedure

The study protocol was approved by the IRB committee of Pusan National University Yangsan Hospital (IRB No. 03-2016-017). When the patients needed IV infusion therapy, the staff nurses inserted 22gauge IV catheter, and explained the purpose and method to the patients or LARs. When they agreed to participate in the study, we obtained written informed consents. And then, we attached electrodes near the IV catheter site at both sides to apply the alternating current and collecting the voltage, and took initial measurement one impedance parameter (Z, *R*, *X*_*C*_, *C*_*m*_) as a function of frequency during infusing an IV solution at the rate of around 60 drops per min. While infusing the IV solution into the subject’s vein, an AC was applied to the electrodes with varying frequencies. To reduce the measurement error, we marked the electrode sites after taking initial measurement and removing the electrodes.

The staff nurses examined the tissue surrounding IV catheter site every shift to identify the signs and symptoms of infiltration such as swelling, redness, and/or discomfort [17] according to their practice manual. When the infiltration was suspected, they quickly reported to a study assistant staying in the hospital to make additional measurement before removing the IV catheter at the same electrode site as at the initial measurement. The impedance parameters measured were kept in the multi-channel impedance measuring instrument automatically and transferred to a PC in text file for data storage and analysis.

**Fig 1:**
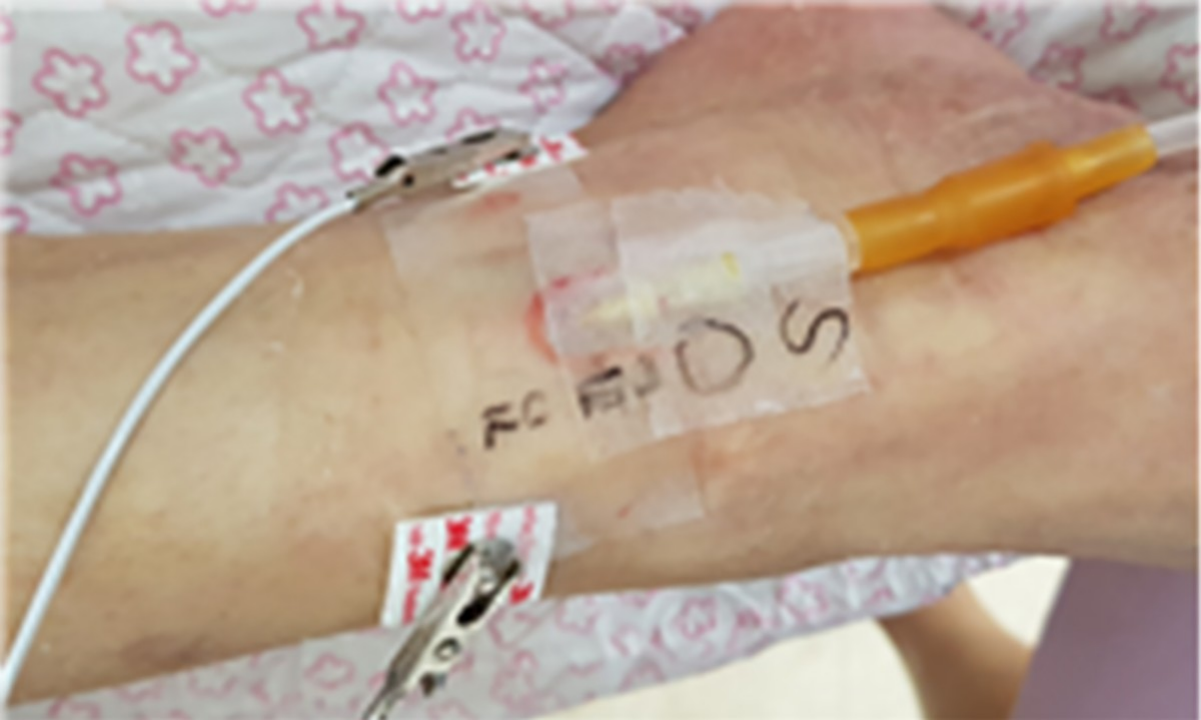
Electrode placement for impedance measurement on the patient's ankle.

Fig 1 shows the electrode placement for impedance measurements in the subject’s ankle.

### Data analysis

Data processing was performed using Excel 2016 (Microsoft Corporation, Redmond, Wash., USA) and the data were graphically analyzed using OriginPro 9.0.0 (OriginLab Corporation, Northampton, Massachusetts, USA).

## Results

### Resistance (*R*) as a function of frequency (*f*)

**Fig 2:**
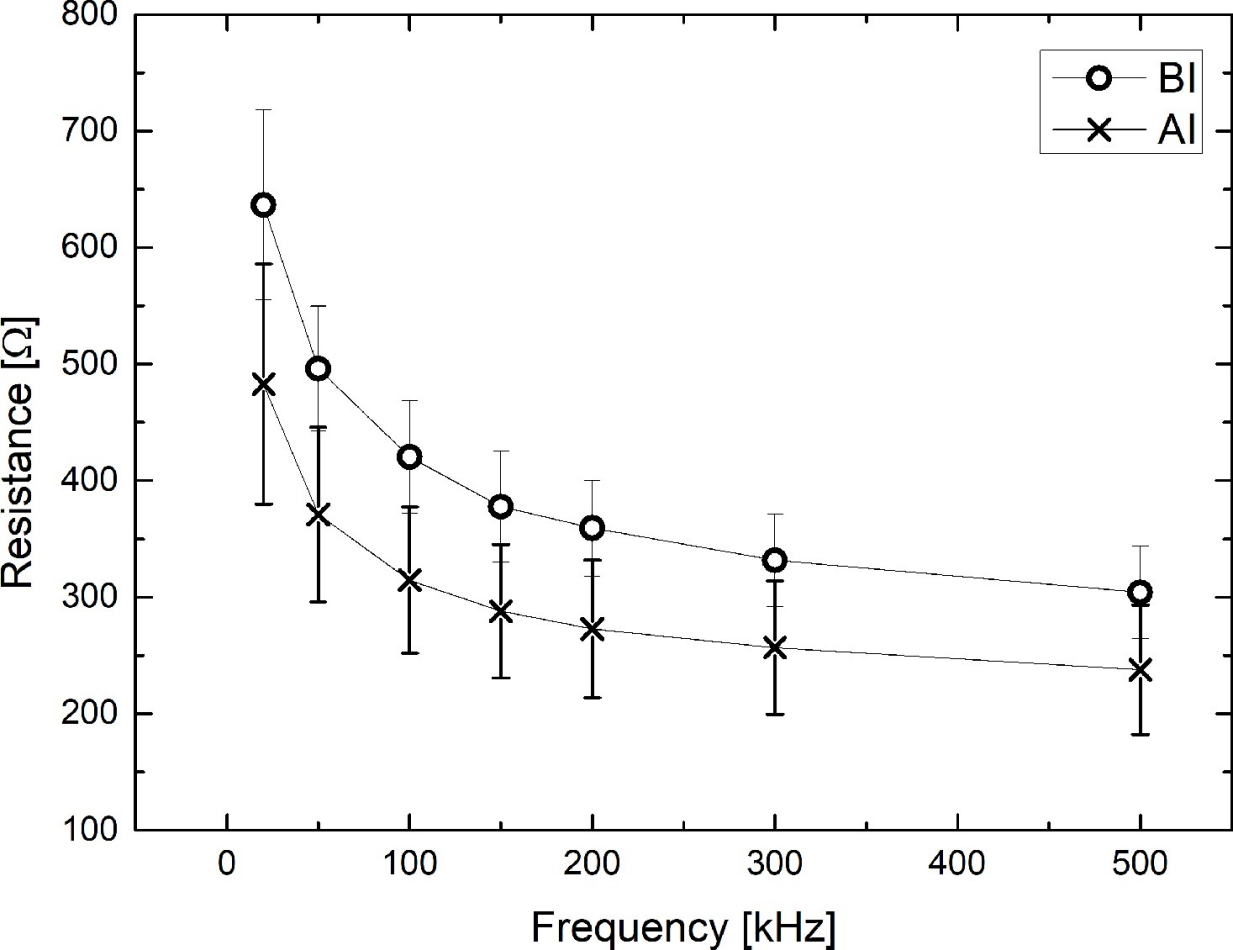
Resistance as a function of frequency during infusion IV solution into the vein. Circle (O) represents the resistance measured before infiltration (BI) and cross (×) represents the resistance measured after infiltration (AI).

Fig 2 shows the mean and standard deviation of the resistance (*R*) as a function of frequency before and after infiltration. Resistance decreased almost inversely with frequency, with resistances showing a significant difference before and after infiltration. While the difference in resistance before and after infiltration was observed clearly, the standard deviation was relatively large. For example, at 20 kHz, the resistances were 643.6±124.1 [Ω] before infiltration and 481.2±114.3 [Ω] after infiltration which corresponded to a 25.2% decrease in resistance. At 50 kHz, the resistances were 498.2±79.3 [Ω] before infiltration and 369.4±85.6 [Ω] after infiltration which corresponded to a 25.8% decrease in resistance.

### Reactance (*X*_*C*_) as a function of frequency (*f*)

**Fig 3:**
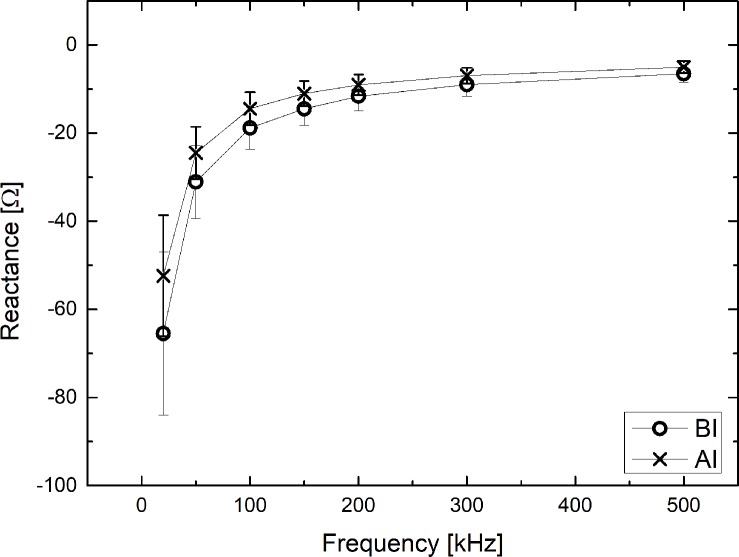
Reactance as a function of frequency before and after infiltration.

Fig 3 shows the reactance (*X*_*C*_) of the cell membrane as a function of frequency before and after infiltration. The absolute magnitude of the reactance decreased in the applied frequency range. In addition, the absolute magnitude of the reactance after infiltration becomes smaller than before infiltration. For example, at 20 kHz, the measured reactance was -65.5±18.5 [Ω] before infiltration and -52.4±13.7 [Ω] after infiltration, corresponding to a 20.0% decrease in reactance. At 50 kHz, the measured reactance was -31.1±8.3 [Ω] before infiltration and -24.5±5.9 [Ω] after infiltration, corresponding to a 21.2% decrease in reactance.

### Resistance (*R*) versus reactance (*X*_*C*_)

**Fig 4:**
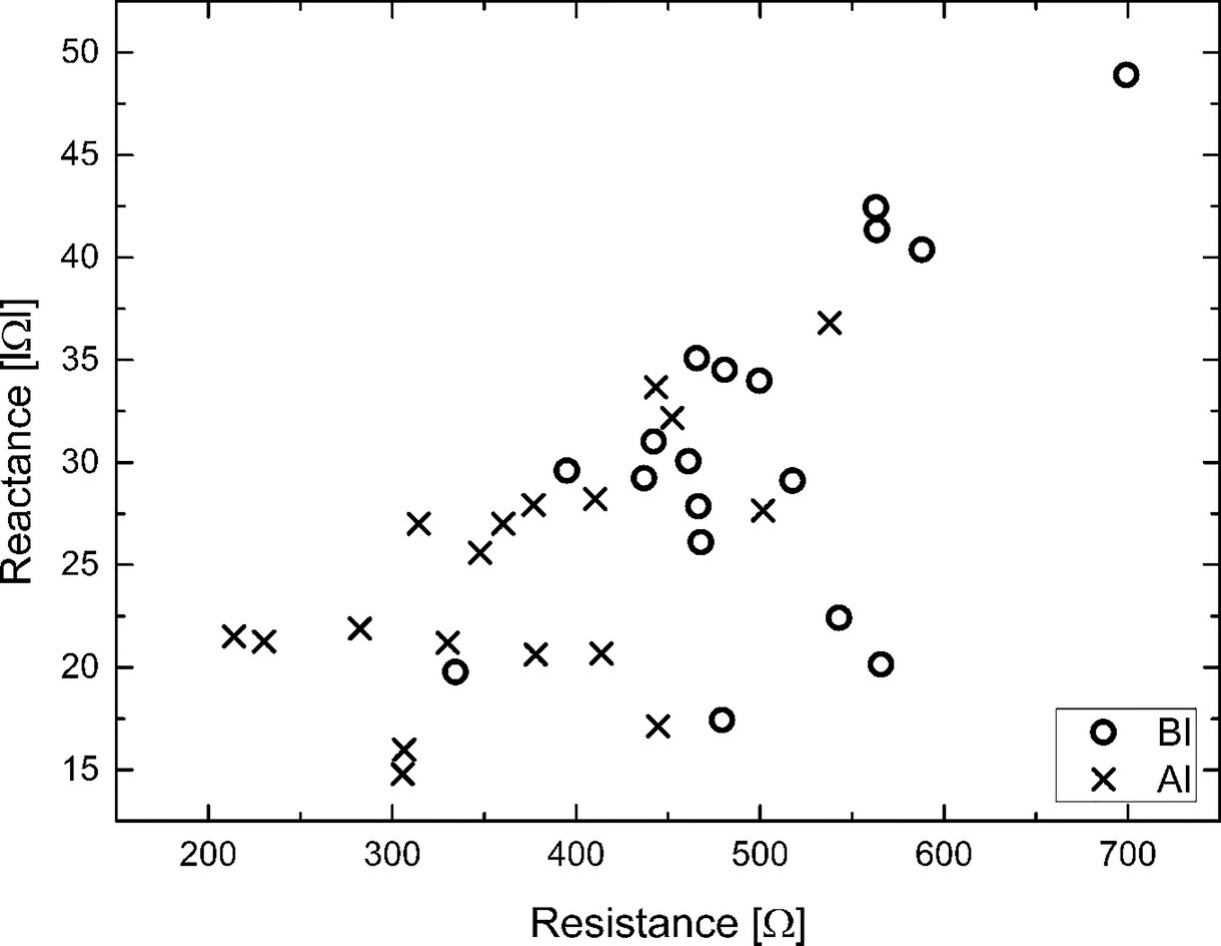
Reactance versus resistance before and after infiltration.

Fig 4 shows the relationship between absolute magnitude of resistance (*R*) and reactance (*X*_*C*_) before and after infiltration at 50 kHz. Compared to the values measured before infiltration, both *R* and *Xc* decreased after infiltration. The positions of *R* and *Xc* mainly distributed in the first quadrant before infiltration have shifted to the third quadrant.

### Capacitance (*Cm*) of cell membrane as a function of frequency (*f*)

**Fig 5:**
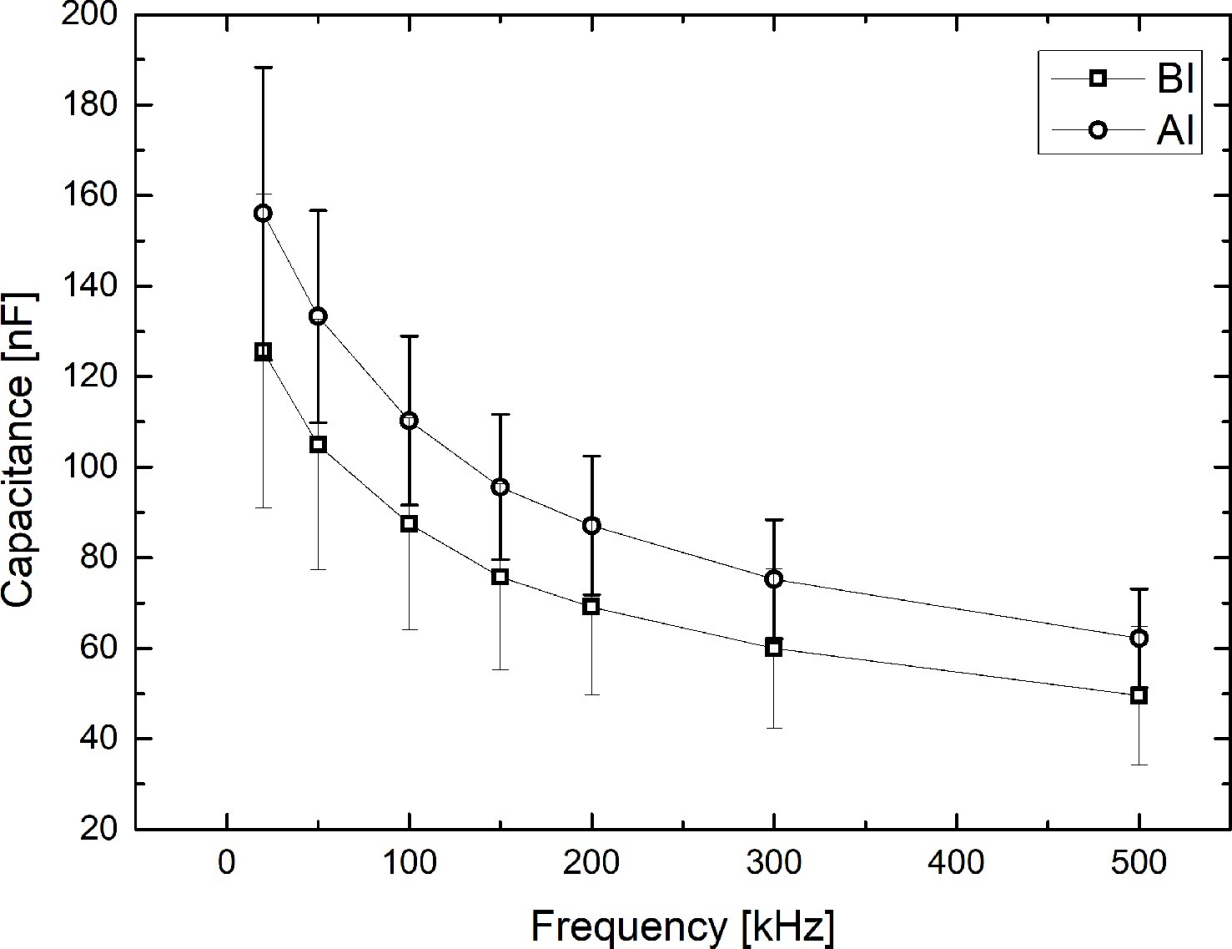
Capacitance of cell membrane before and after infiltration.

Fig 5 shows the capacitance of the cell membrane as a function of frequency. The capacitance of the cell membrane decreases almost inversely as frequency increases. The capacitance of the cell membrane after infiltration increases in the 20–500 kHz frequency range. At 50 kHz, the capacitance of the cell membrane after infiltration increased by 28.0% compared to the capacitance before infiltration.

## Discussion

Impedance indicators have been proposed to detect detection of infiltration of IV solutions that accumulate in the extracellular fluid (ECF) and affect the cell membrane function. In previous experiments that studied infiltration in the rabbit's ears and the human’s forearms, impedance parameters showed a significant difference within 2–3 minutes after infiltration in rabbits, whereas impedance parameters gradually changed over 15 minutes after infiltration in humans [18]. In this human study, impedance parameters before and after infiltration in hospitalized subjects were analyzed using BIA, and infiltration phenomena were quantitatively explained by using infiltration mechanism and equivalent circuit of human cells. Impedance parameters (*R*, *X*_*C*_, *C*_*m*_) showed significant differences in the impedance parameters before and after infiltration.

After infiltration, a reduction in resistance was significantly observed at all frequencies. This indicates that the IV solution flowing from the vein due to infiltration accumulates in the ECF (including interstitial fluid) [19,20]. The large standard deviations shown in Fig 2 are thought to be caused not only by the locations of the forearms, upper arms, heels, hands, legs, feet and thighs (listed in decreasing order of frequency of infiltration) but also the size of infiltration. When AC with a frequency of 20 kHz was used, the resistance was relatively high since AC of frequency of 20 kHz does not pass through the cell membrane, and decreased with increasing frequency.

For reactance, the absolute magnitude of the reactance decreased in the applied frequency range. This is caused by the blood components released from the venous blood vessels in the infiltration, which trigger the cell membranes to aggregate in a linear/ parallel manner, so that the capacitance of the cell membrane increases, and thus the absolute magnitude of the reactance decreases, approaching zero [21]. The IV solution and blood components were absorbed into the cell membrane due to infiltration, critically reducing the cell membrane’s ability to slow down the electric current. Since change in reactance reflects the function of the cell membrane, it is possible to detect a variation in skin or subcutaneous tissue (or necrosis) due to side effects of drug-induced infiltration. Important information about tissue necrosis that occur in neonates with thin blood vessels or in patients experiencing frequent infiltration can be obtained using this phenomenon.

For resistance versus reactance, the results of this measurement are in good agreement with Nescolarde’s study that measured bioelectrical impedance for injuries caused in soccer players' lower extremity muscles [22]. As one recovers from damage caused by infiltration, resistance and reactance are expected to move toward the upper right direction. For instance, localized bio-impedance was used to assess injuries to lower body muscles by measuring soft tissue hydration and cell membrane integrity. Impedance measurements of edema in the calf of the athlete showed that the position of *R*-*Xc* shifted to the lower left according to the severity of injury and to the upper right during the recovery [22]. Compared to non-injury values, *R*, and *Xc* decreased with increasing severity of injury: grade III (23.1% and 45.1%), grade II (20.6% and 31.6%) and grade I (11.9% and 23.5%). These results indicate that a decrease in *R* reflects local body fluid accumulation, and a reduction in *Xc* indicates significant side effects due to compromised cell membrane integrity [23].

The capacitance of the cell membrane after infiltration increased, which may be due to the absorption of IV solution and blood components (red blood cells, white blood cells, platelets, etc.) from the vein into the surrounding cell membranes. In our previous experiments [14], the capacitance of the cell membrane was measured as a function of time up to 25minutes post initiation of infiltration into the veins of the rabbit's ear. The capacitance of cell membranes in rabbit’s ear increased markedly during infiltration and remained almost constant after infiltration. Capacitance of the cell membrane decreases almost inversely as frequency increases. Body capacitance represents the absolute amount of energy storage in the body due to intact cellular membranes. High capacitance indicates that the human body stores energy efficiently, while low capacitance indicates that the cell is inefficient. When a low-frequency current is applied, the cell membrane mainly acts as a resistor. Conversely, when a high-frequency current is applied, the cell membrane behaves primarily as a capacitor [21].

To our knowledge, this study is one of the few studies to identify the relationship between impedance parameters and infiltration. And, it is meaningful to suggest bioelectrical impedance as an effective method for detection of infiltration. That is, we can assume that infiltration may occurred when the resistance (R) and the absolute magnitude of the reactance (*X*_*C*_) decrease, and body capacitance (Cm) increase compared to baseline measure.

However, careful interpretation of the results from the study is required as this study has the following several limitations. First, there is limitation in generalizability because of small sample size with old ages in a single long-term care hospital. Second, the staff nurses inspected the occurrence of infiltration in a regular manner, and measured the parameters at infiltration as quickly as possible. However, there might have a gap between real and detected time of infiltration occurrence, and the parameters at infiltration may not represent them right after the infiltration occurrence. Third, we did not measure the subject’s body composition such as weight and body mass index. However, it may not influence the results because we measured the parameters for the same subjects repeatedly and their body composition is hardly changed for short time between before and after measurement (mean IV day was 1.47 days). Fourth, there were changes in the type of infusate from Combiflex inj. 1000ml (JW Pharmaceutical, Korea) to normal saline or Hartmann’s dextrose at initial and additional measurement for two subjects. It can make a difference for impedance parameters if a conductive (e.g., saline, balanced crystalloid solution) or a non-conductive (e.g., aqua, glucose) solution. As Combiflex inj. 1000ml contains amino acids, electrolytes, emulsion and glucose, changes in the type of infusate may not have a big influence on the results. Fifth, the distance between 2 electrodes is not controlled and it may affect the impedance measurement because the magnitude of the impedance values is inversely proportional to the distance between the electrodes. However, we marked the electrode site at initial measurement, and use the same site for additional measurement. As this study compared the changes in impedance parameters before and after infiltration, the fail to control the distance between electrodes may not influence on the results. Lastly, as we adopted observational study not clinical trial due to ethical issue. we could not control the IV catheter sites and the size of infiltration, which may influence the large standard deviation of the resistance with frequency.

## Conclusions

The resistance (*R*) after infiltration significantly decreased compared to the initial resistance, the absolute magnitude of the reactance (*X*_*C*_) decreased after infiltration, and the data points plotted in the *R-X*_*C*_ graph were distributed on the upper right before infiltration but on the lower left after infiltration. And, body capacitance of the cell membrane decreased almost inversely as frequency increased after infiltration. Our findings suggest that bioelectrical impedance is an effective method for detection of infiltration in a noninvasive and quantitative manner. Repeated studies with different age groups of subjects are recommended to increase the generalizability of the results. And we also recommend further studies to identify the smallest infiltration size that impedance parameters can detect the infiltration, which needs to confirmed whether impedance parameter can use a method for early detection of infiltration.

## Supporting information

S1 File (XLSX)
